# The Effect of Social Capital on Environmental Pollution in China—Suppression or Promotion?

**DOI:** 10.3390/ijerph17249459

**Published:** 2020-12-17

**Authors:** Yuxin Wang, Jinping Xiong, Wenlong Li, Ming Na, Mei Yao

**Affiliations:** 1 School of Economics, Hefei University of Technology, Hefei 230601, China; xiongjinpingjp@163.com (J.X.); lwl18856316518@163.com (W.L.); asterhfut@163.com (M.N.); 2 School of Mathematics, Hefei University of Technology, Hefei 230601, China; ymwalzn@163.com

**Keywords:** social capital, environmental quality, governance effect, China, environmental pollution, collective action

## Abstract

The 19th National Congress of the Communist Party of China clearly regards the prevention and control of environmental pollution as one of the “three key battles” to build a well-off society. This paper analyzes the relationship between social capital and environmental pollution from both theoretical and empirical perspectives. From the theoretical perspective, social capital has two opposite effects on environmental pollution: the suppression and the promotion. The former indicates that the improvement of social capital level reduces the cost of residents’ boycott to pollution and reduces pollution emissions, while the latter manifests that the improvement of social capital worsens environmental pollution due to the ability of polluting enterprises to withstand residents’ boycott. Based on the panel data of China from 2008 to 2016, the empirical results show that social capital has inverted U-shaped characteristics on environmental pollution. Low level of social capital will increase pollution emissions and only when social capital reaches a certain level can it be beneficial to environmental protection. This paper attempts to better understand the functions of social capital in environmental governance and provides constructive proposals on how to exert the governance role of social capital on environmental protection for policy makers. Regions with higher levels of social capital should exert the suppression effect of social capital and regions with low levels should focus on improving the level of social capital, while formal regulation means shall be adopted to control pollution.

## 1. Introduction

Over the past 40 years of reform and opening up, China’s economy has been growing at an astonishing rate of 10% annually. However, the environmental pollution is becoming more and more serious. According to the China Ecological and Environmental Status Bulletin, 217 out of the 338 prefecture-level cities in China exceeded the standard of ambient air quality in 2018 and 338 cities suffered from 1899 days of severe pollution and 822 days of serious pollution. In 2017, 66.6% of the 5100 groundwater quality monitoring points had poor or extremely poor quality. In addition, China’s EPI (Environmental Performance Index) score in 2020 was 37.3, ranking 120th out of 180 countries [1]. From 1988 to 2010, the cost of environmental pollution in China was estimated to account for 8% to 10% of real GDP [2].

It is urgent to improve environmental quality. The Fifth Plenary Session of the 18th Central Committee of CPC clearly put forward the “13th Five-Year Plan” to implement the concepts of innovative, coordinated, green, open and inclusive development. The report of the 19th National Congress put forward the environmental development strategy of “Lucid waters and lush mountains are invaluable assets.” All these show that the Chinese government will firmly carry out the green development strategy and the most stringent environment protection policies.

In recent years, the Chinese government has made many policies of environmental governance, which can be divided into two ways—administrative directives and market incentives. Among these, administrative directives can barely achieve the goal of pollution control and the policy effect far from satisfactory. The pollution charge system based on market incentives becomes the main means of environmental governance. However, the charge system is just a palliative, because as long as the output is higher than the input, enterprises will not stop polluting and the motivation alienation of local government also weakens the effect of charge system [3,4]. Therefore, the exploration of effective environmental governance model becomes the key.

From the successful experience in developed countries, there are other policy options besides administrative directives and market incentives. The ideal way is to form a social norm of environmental protection and to rely on the informal mechanisms to control environmental pollution [5,6,7], which is an important supplement to administrative directive and market incentive. Compared with the formal governance, the informal mechanism does not rely on legal or administrative forces but makes polluting enterprises correct their bad behaviors and trigger the guiding role of social norm through moral and cultural pressures. Members of society take the initiative to investment in the polluting enterprises, consume fewer products made by the polluting enterprises and increase boycotts against pollution emissions, which help reduce pollution at source. So, will this informal governance mechanism based on social capital be effective for China? If effective, how should the government lead the positive role? These are the questions this article will answer.

The concept of social capital originally evolved from the concept of “capital” in economics. In the 1970s, the economist Glenn Loury and sociologist Ivan Light used the term “social capital” to describe the problem of inner-city economic development, which was the first time the concept introduced to the field of social sciences [8]. They believe that social capital is one of many resources that exist in the social organization of family relations and communities. The earliest systematic discourse of social capital theory was proposed by French sociologist Pierre Bourdieu. He formalized the concept of social capital and defined it as a collection of actual or potential resources composed of mutually acquiesced or acknowledged relationships that were more or less institutionalized [9]. Robert D. Putnam’s discussion brought widespread attention to social capital. His definition of social capital is “the characteristics of social organizations that can improve social efficiency by promoting coordinated actions, such as trust, norms and networks” [10], which is widely accepted. The core proposition of the social capital theory is that the network of relationships creates a valuable resource for solving social problems and improving social efficiency. A large number of studies have proved that social capital has an important impact on economic growth, public health and environmental governance [11,12].

In recent years, the impact of social capital on environmental pollution has become a hot spot of scholars all over the world, including China but the conclusions differ a lot. Some researchers believe that social capital is conducive to environmental governance. Keene and Deller [13] used county-data in the United States to study the inhibition of social capital on PM_2.5_ concentration. There is a significant positive correlation between social capital and environmental governance performance and the improvement of social capital can lead to the improvement of environmental protection and the reduction of energy consumption [14,15]. The improvement of social capital contributes to rural environmental governance and cultivating social capital in rural areas is the future direction of rural environmental governance [16,17]. Social capital has a significant positive impact on the rural environment, farmers’ ecological behaviors and domestic waste disposal [18,19,20,21].

Some studies suggest that the impact of social capital on environmental governance is not positive but exacerbates environmental pollution. Social capital governance mechanisms are not necessarily conducive to the governance and protection of the rural ecological environment [22]. The normative dimension of social capital has a significant negative correlation with environmental impact in rural areas [23] and low stock of social capital has a greater negative impact on rural environmental governance [24]. In addition, some studies show that there may be a nonlinear relationship between social capital and pollution. With the increase in the level of social capital, the impact of social capital on pollution presents a trend of first promoting and then suppressing, that is, the impact curve presents an inverted U-shape [25,26].

Most of these studies are based on empirical studies or case studies. Further researches need the explanation and support of theoretical models and it is necessary to clarify the path of social capital’s impact on pollution and the role of different types of social capital. This paper constructs a mathematical model of social capital affecting environmental pollution, analyzes the logical relationship between social capital and pollution from the theoretical perspective. Also, it tests the quantitative relationship by using 2008–2016 provincial panel data and expounds the functional approaches to pollution governance by social capital, which attempts to clarify the dilemma of environmental problem in China from the perspective of social capital. As a matter of fact, no government in any country can possess sufficient administrative resources to supervise all enterprises and individuals who violate the regulations. Therefore, the study of the impact of social capital on environmental pollution not only has an important theoretical contribution but also has a strong practical significance.

The possible marginal contribution of this paper is reflected in three aspects: First, the existing studies mostly verify the relationship between social capital and environmental pollution from the empirical perspective, without indicating the specific impact mechanism. Second, most empirical studies only focus on whether social capital can affect environmental pollution but do not explain what kind of social capital works. Based on the three dimensions of trust, network and norm, this paper measures the social capital at the provincial level, which can better reflect the impact of different social capital and help to understand the heterogeneity of the effect. Third, this paper not only depicts the mechanism of social capital on environmental pollution in theory but also empirically examines the inverted U-shaped impact of social capital on environmental pollution in China, which is a useful supplement to the literature on social capital and environmental pollution and expands the understanding of the function of social capital in environmental governance in transitional countries. Besides, the research results have strong policy significance in that the theoretical explanation and empirical evidence of this paper help us understand the impact of social capital and provide decision-making reference for the formulation of environmental governance policies in different regions of China with different levels of social capita.

The rest of the paper is structured as follows: Section 2 constructs the theoretical model and proposes hypotheses; Section 3 presents the data and methods to verify hypothesis; Section 4 provides results and discussion; Section 5 concludes the paper and gives some policy advice.

## 2. Theoretical Models and Hypothesis

The main argument for the impact of social capital on environment is whether it can influence pollution. If so, then it is important to explore the direction of the influence. Most of the literature in this field focus on empirical analysis and few focus on theoretical study of the impact mechanism. This hinders the explanation of the impact path of social capital. So, we refer to Copeland and Taylor’s analysis of the relationship between export trade and pollution and build a mathematical model to explore how social capital influences pollution when inhibition effect occurs.

By constructing a general equilibrium model of firms and consumers, Copeland and Taylor theoretically analyzed the mechanism of export trade on environmental pollution. Based on their framework, we introduce the impact of social capital on the behavior of enterprises and residents into the model to analyze the impact of social capital on pollution emission. In the Section 2.1 we will elaborate on the assumptions and constructions. In Section 2.2 and Section 2.3, we will analyze the impact of social capital on pollution emission and propose hypotheses.

### 2.1. Model Assumptions

As in Copeland and Taylor’s work, a given enterprise produces product *Y*, using two primary factors, capital (*K*) and labor (*L*). Production of product *Y* generates pollution emissions (*Z*). If the enterprise do not undertake abatement, each unit of output generates one unit of pollution and that output of *Y* is given by *F*(*K*,*L*), where *F*(·) is increasing, concave and linearly homogeneous [27]. If the enterprise does not reduce pollution, then the production function of product *Y* is:

(1) $$Y=Z^{\alpha }{\left[F(K,L)\right]}^{1-\alpha },$$

where *α* ∈ (0,1). Pollution is a by-product and can also be regarded as an input to product *Y*. If governments regulate pollution, the enterprise face a tax *τ* for each unit of emissions that they release and the market price of product *Y* is the given *P*. Interest rates and wages are determined by the external market. Then, when there is no boycott to pollution emissions, the marginal benefits of the enterprise is:

(2) $$\frac{\partial \pi }{\partial Z}=\frac{P\alpha {\left[F(K,L)\right]}^{1-\alpha }}{Z^{1-\alpha }}-\tau .$$

This is a downward sloping curve.

Suppose that residents are all homogenous and their utility function is independent of income and environmental quality. *h*(·) is a concave function that increases incrementally, which describes the harm caused by pollution to individual resident, determined by *Z* [28].

Then, without boycott, the marginal cost caused by pollution to residents is:

(3) $$-\frac{\partial V}{\partial Z}=h^{\prime }(Z).$$

This is an upward sloping curve.

Our model is basically consistent with Copeland and Taylor’s framework by far. Next, we introduce the resident’s boycott into the model to analyze the impact of social capital on enterprise’s pollution emissions.

When the pollution is serious, the increase of pollution emission by enterprises may cause the boycott of residents. We define the intensity of boycott as *boycott*. The cost of boycott corresponding to a certain *boycott* is defined as *Cboycott*. In the initial stage of the collective action, information about boycotts is often disseminated only among a small group of elite activists. A large number of start-up costs are needed, including the cost of the organization, publicity and negotiation and the cost of per residents’ giving up working hours and so forth. When the boycott expanded to a certain extent, the appeal of the elite and the involvement of the media led to a rapid increase in the scale of the boycott, reducing the cost of raising intensity [29,30]. So, we assume that the two-partial derivative of *Cboycott* to *boycott* is negative.

We define the level of social capital as *sci*, which consists of norms, networks and trust [10]. Relationships and networks can influence residents’ organizational participation and increase their efficiency in collective actions, such as responding to public health events [31,32]. Rich social capital can enhance residents’ awareness of justice, such as environmental protection and provide rich channels for residents to voice their opinions [33,34]. So, we assume that the higher the level of social capital is, the easier to initiate a boycott and the lower the cost of boycott.

The boycotted costs which enterprises are faced with are defined as *Cboycotted*, including potential production shutdown cost, restructuring cost and negotiation cost and so forth. When the boycott intensity is low, it is easier to negotiate and the possibility of forced dismounting or rectification of production projects is relatively low. But with the increase of the intensity, especially after the attention of the media and the government has been aroused, the possibility that production projects will eventually be forced to dismount or rectify tends to rise and the risk and cost of enterprises tend to rise as well [29]. So we assume that the two-partial derivative of *Cboycotted* to *boycott* is positive.

Residents’ trust in society will reduce their awareness of social conflict, which will have a negative impact on the incidence of residents’ resistance to defend their rights [35]. When residents have trust in the government and abide by the moral and legal norm, enterprises have efficiency in negotiation and communication between government and residents by rich channels and connections, lower the possibility of violent group conflicts [30]. Therefore, it is assumed that social capital reduces the possibility of intense group conflicts and reduces the risks of polluting project, that is, it reduces the cost of the enterprise due to the boycott.

### 2.2. Hypothesis 1

When *Cboycotted* were lower than the marginal benefit partial ∂*π*/∂*Z* of increased pollution emissions, enterprises would choose to bear the boycotted cost and increase pollution emission. Let *bcted* be the lowest boycott success intensity that makes the enterprises just willing to stop increasing emissions, then it should satisfy:

(4) $$Cboycotted(bcted,sci)=\frac{\partial \pi }{\partial Z}=\frac{P\alpha {\left[F(K,L)\right]}^{1-\alpha }}{Z^{1-\alpha }}-\tau .$$

Obviously, *bcted-Z* is an oblique downward curve.

Let *bct* be the highest boycott intensity of residents. When the intensity is higher than *bct*, the cost of boycott is higher than that of increasing pollution and the residents are not willing to initiate boycott, so *bct* is satisfied

(5) $$Cboycott(bct,sci)=-\frac{\partial V}{\partial Z}=h^{\prime }(Z).$$

Clearly *bct-Z* is an oblique upward curve.

Let expressions (4) and (5) intersect at points (Z*, boycott*).

**Figure 1:**
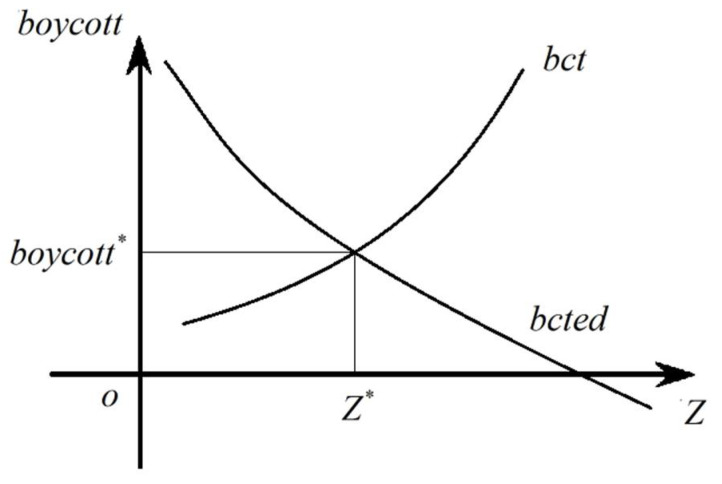
Decision to Equalize Emissions. Note: (Z*, boycott*) is the pollution emission of enterprises and the intensity of residents’ boycott at the equilibrium point.

If *boycott* > *bct*, the cost of choosing to tolerate pollution is lower than the cost of boycott and residents will reduce the intensity of boycott;

If *boycott* < *bcted*, the boycott intensity is not enough to make enterprises give up increasing pollution emissions and residents will increase the intensity;

Thus, when *bcted* > *bct*, residents will not resist the increase of pollution emissions and when *bct* > *bcted*, residents will resist with the intensity of *bcted*. That is, before the pollution emissions reach Z*, enterprises will not face the boycott and after the pollution emissions reach Z*, enterprises will face the boycott with intensity of *bcted*, forcing enterprises to set the level of pollution emissions at Z*. So, (Z*, boycott*) is the equilibrium solution of the model, as shown in Figure 1.

When the level of social capital increases, the *bcted-Z* and *bct-Z* curves of Equations (4) and (5) will move upward, so when the level of social capital increases, there are two opposite effects on the equilibrium point (pollution emission level). One is the promoting effect, the improvement of social capital brings the ability of polluting enterprises to withstand the boycott of residents, the *bcted-Z* curve moves up, the equilibrium point moves right and pollution emission increases. The other is the suppressing effect, with the increase of social capital level, the cost of residents to resist pollution is reduced, the *bct-Z* curve is moved up, the equilibrium point is moved left and the pollution emissions are reduced. Here we get the hypothesis 1.

**Hypothesis** **1(H1).** *There are two opposite effects of increasing the level of social capital on the pollution emission, namely, the promoting effect and suppressing effect, while the final effect depends on the relative magnitude of the two.*

### 2.3. Hypothesis 2

Since the impact of social capital on environmental pollution depends on the relative size of the two effects, then what is the condition that the suppression is greater than promotion? This part will discuss this issue.

The equilibrium solution (Z*, boycott*) of the model is the solution of the simultaneous equations of Equations (5) and (6), that is

(6) $$\left\{ \begin{array}{l} Cboycotted(boycott,sci)=\frac{P\alpha {\left[F(K,L)\right]}^{1-\alpha }}{Z^{1-\alpha }}-\tau \\ Cboycott(boycott,sci)=h^{\prime }(Z) \end{array}\right..$$

Take the total differential on both sides and eliminate the *dboycott*, we get:

(7) $$\frac{dZ}{dsci}=\frac{\frac{\partial Cboycotted}{\partial boycott}\frac{\partial Cboycott}{\partial sci}-\frac{\partial Cboycott}{\partial boycott}\frac{\partial Cboycotted}{\partial sci}}{\frac{\partial Cboycotted}{\partial boycott}h^{\prime \prime }(Z)-\frac{\partial Cboycott}{\partial boycott}(\alpha -1)P\alpha {\left[F(K,L)\right]}^{1-\alpha }Z^{\alpha -2}}.$$

When Equation (7) is positive, the increase of social capital will promote pollution and vice versa. The right denominator of Equation (7) is positive and the condition of social capital promoting pollution emission is:

(8) $$-\frac{\frac{\partial Cboycotted}{\partial boycott}}{\frac{\partial Cboycotted}{\partial sci}}<-\frac{\frac{\partial Cboycott}{\partial boycott}}{\frac{\partial Cboycott}{\partial sci}}.$$

Let the combination of all the (*boycott*, *sci*) such that the boycott cost of the enterprise and the boycott cost of the resident are maintained at a certain level in the *sci-boycott* plane and the curve formed by them be “*equal boycotted cost line*” and “*equal boycott cost line*” respectively. On the left and right sides of Equation (8) are the slopes of the “*equal boycotted cost line*” and the “*equal boycott cost line*” respectively and both are positive. Whether the increase of social capital suppress pollution depends on the relative size of the slopes.

**Figure 2:**
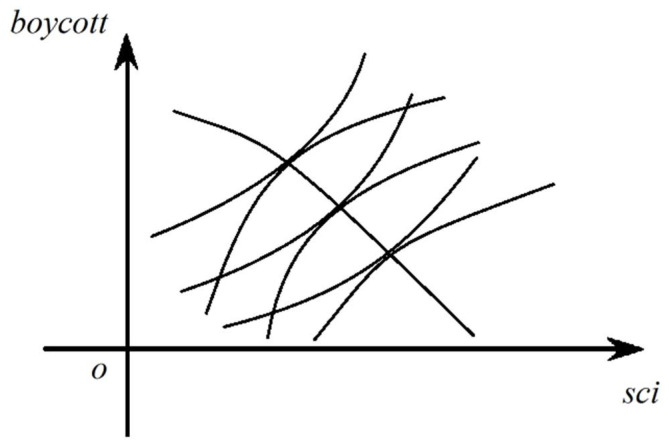
“Equal boycotted cost line,” “Equal boycott cost line” and “Isoclinic”.

For Simplification, assumes that the second-order mixed partial derivatives of *Cboycotted* and *Cboycott* with respect to *boycott* and *sci* are zero. Because the two partial derivatives of *Cboycotted* and *Cboycott* with respect to *boycott* are respectively positive and negative, the slope of “*equal boycotted cost line*” increases with the increase of *boycott*, which is a concave function, while the slope of “*equal boycott cost line*” decreases with the increase of *boycott*, which is a convex function. The “*equal boycotted cost line*” and “*equal boycott cost line*” corresponding to different costs are drawn in the same image, as shown in Figure 2.

There is only one “*equal boycotted cost line*” and one “*equal boycott cost line*” at any point in the quadrant. If the slope of the equal boycott cost curve is greater than the slope of the equal resisted cost curve at this point, it satisfies the condition that the promoting effect is greater than the suppressing effect, that is, social capital promotes pollution at this time and vice versa. If the two curves are tangent at the point, then the two effect cancel each other. The set of points meeting this condition is drawn out to form an oblique downward isoclinic, which is the downward inclined curve in Figure 2. The points at the upper right of the isoclinic satisfy the condition that the suppressing effect is greater than the promoting effect. When the level of social capital increases, as both *bcted-Z* and *bct-Z* curves in Figure 1 move upward, the equilibrium point moves upward and the trajectory of the equilibrium point in Figure 2 moves gradually from the lower left of the isoclinic to the upper right. When social capital reaches a certain level, the suppression effect is dominant. Here we get the hypothesis 2.

**Hypothesis** **2** **(H2).** *With the social capital level increasing from low to high, the impact on pollution will be dominated by promotion effect first and then by suppression effect, that is, the impact of social capital on pollution shows an inverted U-shape.*

## 3. Data, Variables and Model Specification

### 3.1. Data Sources

This paper uses panel data of 30 provinces and cities in China from 2008 to 2016 (Tibet data is missing, so it is excluded). The original data are from Guotai’an database, National Bureau of Statistics, Province Statistical Almanac, China Procuratorial Statistical Almanac and annual report, China Labor Statistical Almanac, China Environmental Statistical Almanac and China Civil Affairs Statistical Almanac.

### 3.2. Variables

The goal of this paper is to study the impact of social capital on environmental pollution at the provincial level. The explanatory variables are environmental pollution indicators, the core explanatory variables are social capital and some variables affecting environmental pollution are controlled. The specific variables and indicators are explained as follows.

#### 3.2.1. Dependent Variable: Environmental Pollution Comprehensive Index

To provide a more comprehensive picture of environmental pollution and to consider the availability of data, the emission of sulfur dioxide, the total amount of waste water and the amount of industrial solid waste are used to study the three pollution sources: gas pollution, liquid pollution and solid pollution. Then the three indexes were synthesized by entropy weight method to get the comprehensive index of environmental pollution [28,36,37,38]. In the robustness test, the emissions of three pollution sources are taken as the explanatory variables and the robustness of the empirical results is further tested.

#### 3.2.2. Core Explanatory Variable: Social Capital

Social capital is the core explanatory variable of this paper and the measurement of social capital is the key. In order to reflect the level of social capital comprehensively, this paper measures social capital at provincial level from three dimensions: social trust, social network and social norm. On the dimension of social trust, we use the data of Zhang and Ke to measure the social trust of provinces and autonomous regions in China [39]. Social network and social norm indicators refer to relevant literature [40,41,42,43,44]. Measuring social networks in terms of information networks, economic networks and interpersonal networks, the Social Network Index is synthesized by entropy weight method, which includes three indexes: the ratio of Internet users to population, the ratio of turnover to GDP and the ratio of passenger traffic to population.

Measuring social norm from three angles: legal norms, social ethics norms and organizational norms. The social norm index is synthesized by the ratio of the number of labor dispute cases accepted to GDP, the ratio of the amount of donation (direct and indirect) to GDP and the ratio of the original insurance premium income to GDP. Finally, we synthesize social trust index, social network index and social norm index by entropy method to get social capital comprehensive index.

#### 3.2.3. Control Variables

Referring to the literature on environmental pollution [28,36,37,38], the following variables were selected as control variables.

Output level (GDP): GDP per capita (flattened to 2008) and its quadratic term; Technological Progress: the impact of technological progress is controlled by the ratio of capital to Labor, in which the stock of material capital is estimated by Zhang [45] using the perpetual inventory method and the whole society invests in fixed assets based on the 2000 prices, the economic depreciation rate is 9.6% and labor is measured by the number of employed people at the end of the year in each region. Structural effect: the proportion of secondary industry economy; Foreign investment: the actual utilization of foreign direct investment (flattened to 2008); Policy elimination effect: the proportion of GDP invested in industrial pollution control; Government size: the proportion of GDP consumed by the government; Quality of government: Number of cases of official crimes (corruption, bribery and malfeasance) as a proportion of the number of people in public administration, social security and social organizations at the end of the year. Population: total population by region at the end of each year. In addition, year dummy variables are added to the control variables to control the year. The definitions and descriptive statistics for all variables are shown in Table 1.

### 3.3. Empirical Model Setting

In order to study the non-linear property of the influence of social capital on environment pollution, the quadratic term of social capital is added to the linear regression and its model equation is

(9) $$\begin{array}{l} pollution_{it}=\alpha_{1}sci_{it}+\beta X_{it}+u_{i}+\lambda_{t}+\varepsilon_{it}\\ pollution_{it}=\alpha_{1}^{\prime }sci_{it}+\alpha_{2}^{\prime }sci_{it}^{2}+\beta^{\prime }X_{it}+u_{i}^{\prime }+\lambda_{t}^{\prime }+\varepsilon_{it}^{\prime } \end{array}$$

Here *sci_it_* is the social capital index, which are social trust (*trust*), social network (*network*), social norm (*norm*) and social capital comprehensive index (*sci*) and *X* represents the relevant control variables.

## 4. Results and Discussion

### 4.1. Impact of Comprehensive Social Capital

Firstly, the regression of the basic model is carried out. Taking 30 provinces and cities in China from 2008 to 2016 as samples and taking the comprehensive index of environmental pollution as the explained variable to measure environmental pollution. Hausman test shows that the fixed effect model is applicable and the two-way fixed effect model is used to control the years and regression is carried out. The results are reported in Table 2.

The regression results show that after controlling a series of environmental pollution-related variables, regional heterogeneity and time effect, the estimated coefficient of social capital is significantly positive and the estimated coefficient of quadratic term of social capital is significantly negative. This shows that the impact of social capital on environmental pollution presents a significant feature of first promoting and then suppressing, that is, the influence curve shows an inverted U-shape. The result is consistent with the prediction given by the theoretical model constructed above and Hypotheses 1 and 2 are verified.

When the level is low, the improvement of social capital will not reduce environmental pollution but lead to the increase of pollution, which has been analyzed in the theoretical model. When social capital reaches a certain level, its improvement will help reduce environmental pollution. At this time, the connection between collectives reduces the cost of collective action. Residents can launch effective boycott against polluting enterprises and enterprises are at greater risk of losses caused by boycott. Therefore, enterprises tend to reduce pollution discharge. In our results, the social capital index is 6.778 at the vertex of the curve, of which 27 provinces and cities are higher than 6.778 in 2016. It shows that the level of social capital in most regions of China is in the latter half of the inverted U-shaped curve. That is to say, the increase of social capital can suppress pollution in most regions.

### 4.2. Impact of Different Types of Social Capital

Social capital is a comprehensive index constructed. To further confirm the relationship between social capital and environmental pollution, we regress different dimensions of social capital, that is, we regress three social capital indicators and their quadratic items respectively. Because the social trust index used in this paper does not change over time, the equation estimation of the social trust index cannot use the fixed effect model but the random effect model and other equations control for annual and regional heterogeneity. The results are reported in Table 3.

The results show that the effect of different types of social capital on environmental pollution has not changed. The influence of social network and social norm on the comprehensive pollution index also shows an inverted U-shape. Both linear and quadratic coefficients are consistent with the direction of the basic model and significant. This suggests that the network dimension of social capital plays a role in environmental regulation. When the level of social network is low, it is easy for polluters and local governments to reach a conspiracy in the absence of public supervision, thus exacerbating environmental pollution [46]. The rich social connections speed up the flow of information in the society, improve the residents’ identification of nepotism [32] and reduce the possibility of collusion between government and enterprises to cause pollution disorder. Rich social network is the basis for launching social environmental protection movement. A high degree of regional contact is conducive to launching collective action and the developed degree of contact network can effectively promote the participation of social groups in public governance including environmental protection action. Besides, the degree of acceptance of law, public morality and organizational norms measures the social capital of normative dimension. At a low level of social norm, the system of administrative instructions and market incentives is vulnerable to damage and the awareness of environmental protection is difficult to spread among groups. When the level of social norms is high, residents tend to voluntarily comply with environmental protection policies, thus increasing the public supervision of enterprises’ pollution behaviors. The influence of trust social capital also presents an inverted U-shape but it is not statistically significant. From a theoretical perspective, trust can improve the efficiency of collective action and is an important basis for the formation of justice consciousness such as environmental protection [14].

### 4.3. Robustness Test

#### 4.3.1. Changing Environmental Pollution Indicators

In order to further test the robustness of the model, the original composition data of the environmental pollution comprehensive index are selected as explanatory variables to verify the stability. The results are shown in Table 4 and Table 5.

According to the results of columns 1–3 in Table 4, the effects of the comprehensive social capital index on the emissions of the three pollutants are also inverted U-shaped, with the linear and quadratic coefficient keeping in the same direction as that of the basic model. The vertex values of the SO_2_ emission curve and the waste water emission curve are higher than the basic model curve, which are 7.658 and 9.719 respectively. This shows that industrial solid waste pollution can get the control effect under the lower level of social capital stock, while air pollution and waste water pollution need higher social capital stock to cross the vertex of negative effect and achieve the positive effect of pollution control.

As shown in columns 4–6 of Table 4 and Table 5, the effects of social trust and social network on SO_2_ emission and waste water emission are inverted U-shape. Combined with the results of regression with only linear items, the effects of social trust and social network on industrial solid waste emission are unidirectional inhibition but the effects of social norm on industrial solid waste emission are not significant. The influence of social norm on three kinds of pollutant emission is not inverted U-shape. In the regression with only linear items, social norm has no significant effect on SO_2_ emission and waste water emission but has one-way restraining effect on solid waste emission. For the emission of industrial solid waste, the regression results with the quadratic terms all do not show U-shaped characteristics. The possible reason is that the social capital index at the vertex of the curve for industrial solid waste is 6.033, while only 12 out of the 270 samples observed in this paper have a social capital index below that value. For industrial solid waste emission, most samples have gone over the vertex of the influence curve and show unidirectional suppression effect, so the inverted U-shape cannot be recognized.

#### 4.3.2. Changing Sample Size: Deleting Municipalities

Considering that the policy, scale and economic status of municipalities are quite different from those of ordinary provinces, we delete municipalities (Beijing, Tianjin, Shanghai and Chongqing) from the samples and use the province sample for regression to further test the robustness of the model. The results are reported in Table 6.

The results show that, after excluding the sample of municipalities, the inverted U-shaped characteristics of the comprehensive social capital index on environmental pollution are still significant and the *sci* value at the vertex of the comprehensive environmental pollution curve is 7.238, which has a small change compared with the basic model.

### 4.4. Endogenous Problems

The existing literature rarely discusses endogeneity of social capital and environmental pollution. In order to overcome possible endogenous problems, the social capital indicators’ lagging terms of the first period and lagging terms of the second period and quadratic terms are introduced as instrument variable. Social trust index is not changed with time and cannot be treated with lag, so only social network, social norm and comprehensive social capital index are re-estimated by panel instrumental variables.

It can be seen from Table 7 that the impact of different types of social capital and total social capital on environmental pollution shows a significant inverted U-shaped characteristic and the conclusion is basically the same as the previous conclusion.

## 5. Conclusions

In recent years, China’s environmental pollution control has achieved remarkable results but with China’s economy from the stage of high-speed growth to the stage of high-quality development, China’s development has entered a new era. The prevention and control of environmental pollution as one of the “three key battles” to win a well-off society in an all-round way, will be an important part of the work of the Chinese government. The results show that the influence of social capital on environment pollution has two opposite effects. With the change of the relative size of the two effects at different levels of social capital, the influence of social capital on environmental pollution is an inverted U-shape, which is promoted first and then suppressed. According to the empirical results of this paper, we find that the social capital index at the inflection point is 6.778. In 2016, 27 out of 30 provinces and cities reported a higher level of social capital than 6.778, indicating that the level of social capital in most regions of China is actually in the second half of the inverted U-shaped curve, the improvement of social capital can reduce environmental pollution and social capital can be used as an important supplementary means outside the administrative directives and market incentives.

The conclusions of this paper have significant implications for the mechanism design of environmental governance in China at the present stage, help to correctly understand the impact of social capital and provide decision-making reference for the formulation of environmental governance policies in different regions of China at different levels of social capital.

For the environmental pollution control in regions with high level of social capital, we should vigorously promote the participation of social capital in environmental governance, improve the participation, supervision and feedback mechanism of the masses and social organizations in pollution projects and pollution control and give full play to the environmental governance effect of social capital.

For regions with low levels of social capital, especially Hainan, Qinghai and Ningxia where the level of social capital is lower than the inflection point, in addition to solving the fundamental problem of improving the social capital participation mechanism, it is also necessary to pay attention to the role of social capital in promoting pollution projects by enterprises. Since the public lacks appropriate channels to participate in pollution control, the government should act as a gatekeeper in environment governance and supervise the pollution behavior of enterprises, instead of allowing enterprises to invest in polluting projects without receiving public feedback.

For all local governments, the level of local social capital should be improved from the perspectives of networks, norms and trust. In terms of social networks, governments should improve the efficiency of information transmission, increase the openness and transparency of administrative operations and use social supervision to reduce collusion between government and enterprises. In terms of social norms, it is necessary to improve the degree of residents’ recognition of environmental protection policies, increase the policy interaction between the government and the public and make enterprises and individuals take the initiative to comply with environmental regulations. In terms of trust, the government should actively promote the concept of environmental protection, encourage residents to report pollution and encourage compliance activities of environmental protection agencies.

All in all, the environmental problems and pressures China is facing are severe, which undoubtedly requires the joint efforts of the whole society, not only the effective supervision of the government, clear law enforcement and focus reports of the media are needed but also the active and extensive participation of the public is important. By strengthening the governance role of social capital, we can provide new ideas and perspectives for the institutional reform of the construction of ecological civilization at this stage and find a new way to effectively govern environmental problems.

## Figures and Tables

**Table 1:** Variable descriptions.

	Variable Symbol	Variable Name	Unit	Mean Value	Standard Deviation
Environmental pollution	epu_so2	Sulfur dioxide emission	Ton	667,895.1	407,591.1
	epu_fspfl	Effluent emission	Ten thousand tons	221,324.6	174,379.1
	epu_gtfwcsl	Amount of industrial solid waste produced	Ten thousand tons	9533.969	8443.114
	pollution	comprehensive pollution index	-	26.54026	14.81424
Social Capital Variables	trust	Social trust index	-	39.32	52.31749
	network	Social network index	-	29.24783	12.72902
	norm	Social norm index	-	13.24474	7.033922
	sci	Social capital index	-	19.0549	18.38683
Control Variables	gdpp	Per capita GDP	Ten thousand yuan per person	4.300916	2.423255
	klrate	Ratio of capital to labor	Ten thousand yuan per person	5.290774	6.312641
	secondary	Proportion of the secondary industry	-	0.4675102	0.081212
	fdi	Foreign direct investment	Ten thousand dollars	681,194.3	711,730.3
	egv_gywrzlzb	Proportion of GDP invested in industrial pollution control	0.01%	14.45034	12.53388
	gov_scale	Size of government	-	0.1445506	0.041521
	gov_corrupt	Government quality	Pieces/million people	2401.41	688.912
	popu	Population	Ten thousand people	4482.019	2684.391

**Table 2:** Regression results of basic model.

Variables	(1) Lnpollution	(2) Lnpollution
lnsci	−0.137 **	0.643 **
	(0.061)	(0.284)
lnsci2		−0.168 ***
		(0.060)
lngdpp	0.440 ***	0.653 ***
	(0.141)	(0.159)
lngdpp2	−0.155 ***	−0.120 ***
	(0.044)	(0.045)
klrate	−0.001	0.001
	(0.007)	(0.007)
secondary	−0.362	−0.858 **
	(0.345)	(0.383)
lnfdi	−0.105 ***	−0.097 ***
	(0.024)	(0.024)
egv_gywrzlzb	0.002 **	0.003 **
	(0.001)	(0.001)
gov_scale	−1.022	−0.654
	(0.673)	(0.675)
lngov_corrupt	0.120 *	0.127 *
	(0.067)	(0.066)
lnpopu	1.250 **	0.788
	(0.518)	(0.536)
Sample size	270	270
R squared	0.972	0.973
Sci value at the vertex	-	6.778

Notes. (1) The symbols *, ** and *** indicate statistical significance at the 10%, 5% and 1% levels, respectively. (2) The estimated results of the coefficients of the year dummy variables and constant terms are omitted in the table. (3) The logarithmic processing is performed by adding “ln” before the variables.

**Table 3:** Regression results of different types of social capital on pollution.

Variables	(1) Lnpollution	(2) Lnpollution	(3) Lnpollution
lntrust	0.052		
	(0.171)		
lntrust2	−0.010		
	(0.023)		
lnnetwork		1.934 ***	
		(0.410)	
lnnetwork2		−0.278 ***	
		(0.062)	
lnnorm			0.574 ***
			(0.189)
lnnorm2			−0.111 ***
			(0.033)
lngdpp	0.535 ***	0.412 ***	0.309 **
	(0.196)	(0.144)	(0.142)
lngdpp2	−0.008	−0.102 **	−0.149 ***
	(0.076)	(0.046)	(0.043)
klrate	−0.013 **	−0.012 *	−0.005
	(0.006)	(0.007)	(0.007)
secondary	2.624 ***	−0.372	−0.193
	(0.334)	(0.342)	(0.336)
lnfdi	−0.126 ***	−0.132 ***	−0.111 ***
	(0.029)	(0.023)	(0.023)
egv_gywrzlzb	0.009 ***	0.002 **	0.002 **
	(0.002)	(0.001)	(0.001)
lngov_corrupt	−0.183 **	0.144 **	0.098
	(0.076)	(0.065)	(0.067)
gov_scale	0.230	−1.050	−1.160 *
	(0.726)	(0.648)	(0.661)
lnpopu	0.883 ***	2.060 ***	1.531 ***
	(0.074)	(0.495)	(0.488)
Sample size	270	270	270
R squared	0.831	0.974	0.973
Sci value at the vertex	13.464	32.408	13.271

Notes. (1) The symbols *, ** and *** indicate statistical significance at the 10%, 5% and 1% levels, respectively. (2) The estimated results of the coefficients of the year dummy variables and constant terms are omitted in the table. (3) The logarithmic processing is performed by adding “ln” before the variables.

**Table 4:** Regression results of environmental pollution indicators.

Variables	(1) Lnepu_so2	(2) Lnepu_fspfl	(3) Lnepu_gtfwcsl	(4) Lnepu_so2	(5) Lnepu_fspfl	(6) Lnepu_gtfwcsl
lnsci	1.140 ***	0.614 ***	0.889 *			
	(0.335)	(0.233)	(0.491)			
lnsci2	−0.280 ***	−0.135 ***	−0.248 **			
	(0.070)	(0.049)	(0.103)			
lntrust				0.600 **	−0.270 **	0.446
				(0.246)	(0.109)	(0.341)
lntrust2				−0.065 *	0.062 ***	−0.100 **
				(0.034)	(0.015)	(0.047)
Control variables	Yes	Yes	Yes	Yes	Yes	Yes
TIme effect	Yes	−	-	Yes	Yes	Yes
Regional effect	Yes	Yes	Yes	Yes	Yes	Yes
R squared	0.978	0.988	0.963	0.784	0.951	0.678
Sample size	270	270	270	270	270	270
Sci value at the vertex	7.658	9.719	6.003	-	-	-

Notes. (1) The symbols *, ** and *** indicate statistical significance at the 10%, 5% and 1% levels, respectively. (2) The estimated results of the coefficients of the year dummy variables and constant terms are omitted in the table. (3) The logarithmic processing is performed by adding “ln” before the variables.

**Table 5:** Regression results of environmental pollution indicators.

Variables	(7) Lnepu_so2	(8) Lnepu_fspfl	(9) Lnepu_gtfwcsl	(10) Lnepu_so2	(11) Lnepu_fspfl	(12) Lnepu_gtfwcsl
lnnetwork	1.449 ***	2.003 ***	1.323 *			
	(0.506)	(0.322)	(0.737)			
lnnetwork2	−0.226 ***	−0.294 ***	−0.174			
	(0.077)	(0.049)	(0.112)			
lnnorm				0.176	0.039	0.522
				(0.233)	(0.158)	(0.332)
lnnorm2				−0.027	−0.013	−0.111 *
				(0.041)	(0.028)	(0.058)
Control variables	Yes	Yes	Yes	Yes	Yes	Yes
TIme effect	Yes	Yes	Yes	Yes	Yes	Yes
Regional effect	Yes	Yes	Yes	Yes	Yes	Yes
R squared	0.977	0.989	0.962	0.976	0.987	0.962
Sample size	270	270	270	270	270	270
Sci value at the vertex	-	-	-	-	-	-

Notes. (1) The symbols * and *** indicate statistical significance at the 10% and 1% levels, respectively. (2) The estimated results of the coefficients of the year dummy variables and constant terms are omitted in the table. (3) The logarithmic processing is performed by adding “ln” before the variables.

**Table 6:** Regression results after deleting municipality.

Variables	(1) Lnpollution	(2) Lnpollution	(3) Lnpollution	(4) Lnpollution
lnsci	1.152 ***			
	(0.405)			
lnsci2	−0.291 ***			
	(0.091)			
lntrust		0.374 **		
		(0.174)		
lntrust2		−0.049 **		
		(0.024)		
lnnetwork			1.748 ***	
			(0.474)	
lnnetwork2			−0.253 ***	
			(0.075)	
lnnorm				0.335 *
				(0.184)
lnnorm2				−0.073 **
				(0.032)
Control variables	Yes	Yes	Yes	Yes
TIme effect	Yes	-	Yes	Yes
Regional effect	Yes	-	Yes	Yes
R squared	234	234	234	234
Sample size	0.976	0.832	0.976	0.976
Sci value at the vertex	7.238	-	-	-

Notes. (1) The symbols *, ** and *** indicate statistical significance at the 10%, 5% and 1% levels, respectively. (2) The estimated results of the coefficients of the year dummy variables and constant terms are omitted in the table. (3) The logarithmic processing is performed by adding “ln” before the variables.

**Table 7:** Two-stage regression results of instrumental variable method.

Variables	(1) Full Sample Lnpollution	(2) Full Sample Lnpollution	(3) Full Sample Lnpollution	(4) Eliminating Municipalities Lnpollution	(5) Eliminating Municipalities Lnpollution	(6) Eliminating Municipalities Lnpollution
lnsci	0.724 **			1.234		
	(0.359)			(0.830)		
lnsci2	−0.248 ***			−0.378 *		
	(0.065)			(0.206)		
lnnetwork		4.328 ***			4.542 ***	
		(0.987)			(1.531)	
lnnetwork2		−0.646 ***			−0.698 ***	
		(0.144)			(0.232)	
lnnorm			1.226 **			1.024 **
			(0.509)			(0.416)
lnnorm2			−0.238 ***			−0.221 ***
			(0.081)			(0.066)
Control variables	Yes	Yes	Yes	Yes	Yes	Yes
TIme effect	Yes	Yes	Yes	Yes	Yes	Yes
Regional effect	Yes	Yes	Yes	Yes	Yes	Yes
R squared	210	210	210	182	182	182
Sample size	0.988	0.981	0.989	0.989	0.982	0.991
Sci value at the vertex	4.305	-	-	5.115	-	-

Notes. (1) The symbols *, ** and *** indicate statistical significance at the 10%, 5% and 1% levels, respectively. (2) The estimated results of the coefficients of the year dummy variables and constant terms are omitted in the table. (3) The logarithmic processing is performed by adding “ln” before the variables.
